# The effect of music on pain management in preterm infants during daily painful procedures: a systematic review and meta-analysis

**DOI:** 10.3389/fped.2024.1351401

**Published:** 2024-02-07

**Authors:** Yiran Ou, Ling Chen, Xinyue Zhu, Tianci Zhang, Ye Zhou, Lu Zou, Yun Gao, Zhenghao Wang, Xiaofeng Zheng

**Affiliations:** ^1^ Department of Endocrinology and Metabolism, Center for Diabetes and Metabolism Research, West China Hospital, Sichuan University, Chengdu, China; ^2^ Institute of Taoism and Religious Culture, Sichuan University, Chengdu, China

**Keywords:** premature infant, music, procedural pain, PIPP, meta-analysis

## Abstract

**Background:**

The present systematic review and meta-analysis of randomized controlled trials (RCTs) was conducted to investigate the effects of music on pain management in preterm neonates during painful procedures.

**Methods:**

The PubMed, Embase, Web of Science, EBSCO and Cochrane Library databases were searched to identify relevant articles published from their inception to September 2023. The study search strategy and all other processes were implemented in accordance with the Preferred Reporting Items for Systematic Reviews and Meta-Analyses (PRISMA) statement.

**Results:**

Four RCTs that satisfied the inclusion criteria were included in this meta-analysis. The music group had significantly lower Premature Infant Pain Profile (PIPP) scores during (RR = −1.21; 95% CI = −2.02–−0.40, *p* = 0.0032) and after painful procedures (RR = −0.65; 95% CI = −1.06–−0.23, *p* = 0.002). The music group showed fewer changes in PIPP scores after invasive operations than did the control group (RR = −2.06; 95% CI −3.16–−0.96; *p* = 0.0002). Moreover, our results showed that music improved oxygen saturation during (RR = 3.04, 95% CI = 1.64–4.44, *p* < 0.0001) and after painful procedures (RR = 3.50, 95% CI = 2.11–4.90, *p* < 0.00001). However, the change in peak heart rate during and after painful procedures was not statistically significant (RR = −12.14; 95% CI = −29.70−5.41 *p* = 0.18; RR = −10.41; 95% CI = −22.72−1.90 *p* = 0.10).

**Conclusion:**

In conclusion, this systematic review demonstrated that music interventions are effective for relieving procedural pain in preterm infants. Our results indicate that music can reduce stress levels and improve blood oxygen saturation. Due to the current limitations, large-scale, prospective RCTs should be performed to validate the present results.

## 1 Introduction

The World Health Organization defines preterm birth as a birth before 37 completed weeks of gestation or fewer than 259 days after the first day of the woman's last menstrual period. In 2020, an estimated 13.4 million babies were born preterm, accounting for 10% of all live births worldwide. In 2020, the preterm birth rate varied widely, from 4%–16% among different countries (1). In 2019, 5.30 million children younger than 5 years died—17% of whom died due to preterm birth complications (2)—and most survivors were hospitalized in the neonatal intensive care unit (NICU), with a length of hospitalization ranging from a few days to months. Hospitalized neonates experience acute episodic pain every day, including pain caused by heel lancing, naso- and endotracheal suctioning, venipuncture, arterial puncture, tracheal intubation, tracheal extubation, peripherally inserted central venous catheter (PICC) placement, retinopathy of prematurity (ROP) examination and other special invasive operations according to their individual clinical requirements (3). Most preterm infants require hospitalization in the NICU, where pain exposure is even more prevalent. According to a European multicenter study, many painful and stressful procedures were performed in the NICU, and the majority of them were performed without analgesic measures (4). According to the traditional definition, pain is an unpleasant sensory and emotional experience that leads to systemic physiological changes. However, this definition strongly relies on patients to describe their pain, establishing the primacy of self-reports as the “gold standard” for newborns (who are nonverbal) and proposing that pain in newborns be classified as acute episodic, acute recurrent, prolonged, persistent or chronic pain (5). Based on a global, four-stage consensus and validation, acute episodic pain is described as a painful response to a procedure or an event. An episode is defined as a single event or a sequence of events related to a procedure (6). Although newborns are unable to describe their pain, physiological and behavioral responses are difficult to ignore. The physiological changes include increased heart rate and blood pressure, decreased blood oxygen saturation, fluctuating cortisol levels, and tissue damage (7). The physiological changes include increased heart rate and blood pressure, decreased blood oxygen saturation, fluctuating cortisol levels, and tissue damage (5). In addition to erratic fluctuations in vital signs caused by painful stimuli, the long-term adverse effects of painful experiences are of concern. Recent studies have shown that exposure to persistent or recurrent pain or stress in the infancy period has long-term consequences that not only affect brain and behavioral development but also affect all organs, leading to serious morbidity and potential mortality (6, 7). Moreover, preterm infants who survive are more susceptible to prolonged negative effects throughout their lives, such as permanent changes in the endocrine and immune systems and impaired reactivity toward stressful events (8–10). According to the evidence-based clinical practice guidelines for the management of newborn pain, pain management in newborns requires a multidimensional approach that includes environmental, pharmacological and non-pharmacological measures (3). Although pharmacological treatments for neonates are established early and have relatively certain effects, the immature drug metabolism and related negative side effects, such as respiratory depression, apnea, bradycardia and hypotension, are difficult to overlook, especially for premature infants (11). Therefore, there has been growing interest in nonpharmacological analgesic options such as oral sucrose administration, nonnutritive sucking (NNS), kangaroo care, breastfeeding, and massage (11, 12).

Among all nonpharmacological analgesic options, music has long been used to enhance well-being and reduce pain and suffering. According to the definition put forward by the American Music Therapy Association, music therapy refers to the clinical and evidence-based use of music as a therapeutic intervention to accomplish individualized goals facilitated by a credentialed professional who has completed an approved music therapy program (13). Music therapy has been utilized as a strategy to ameliorate pain in various healthcare settings due to its noninvasiveness, cost effectiveness, reduction in anesthetic dosage and promising analgesic effects. However, the effects of music on pain management in preterm infants during painful procedures from the aspect of pain assessment have not been determined. For newborn patients, especially preterm infants, self-expression is not possible. Therefore, valid and reliable tools for assessing the degree of pain in newborns are needed (14). There are approximately 40 pain assessment instruments available to date, with various evaluation dimensions (15). The different assessments include different items and have been validated for patients with various kinds of pain and ages (16). The Premature Infant Pain Profile (PIPP) was developed by Stevens, Johnston, Petryshen, and Taddio to evaluate preterm infants' pain responses. This scoring system includes seven items: two items that describe baseline characteristics (gestational age and behavioral state); two items that are physiological indicators (heart rate and oxygen saturation); and three items that describe the neonate's facial expression (brow bulge, eye squeeze, and nasolabial furrow); the score for each item ranges from 0 to 3. A PIPP score of 0–6 points indicates mild pain, 7–12 points indicates moderate pain, and 13–21 points indicates severe pain (17). The PIPP is used to evaluate acute procedural pain in preterm and term infants (18). As the instrument takes contextual factors such as gestational age into account, the PIPP is recommended for its ability to judge pain in preterm and even extremely preterm neonates (16).

In addition, new studies with more detailed data and higher evidence levels have been published (19–22). Thus, we performed the present systematic review and meta-analysis of randomized controlled trials (RCTs) to investigate the effects of music on pain management in premature newborns. The results of this investigation may guide future decision-making regarding the use of music for managing pain in premature newborns.

## 2 Materials and methods

This systematic review and meta-analysis followed the guidelines of the Preferred Reporting Items for Systematic Reviews and Meta-analysis (PRISMA) statement and the Cochrane Handbook for Systematic Reviews of Interventions. Ethical approval and patient consent were not required because all analyses were based on previously published studies.

### 2.1 Literature search and selection criteria

We systematically searched several databases, including PubMed, Embase, the Web of Science, EBSCO and the Cochrane Library, from inception to September 2023. The research strategy was as follows: ((((((((((((((((((((“Music”[Mesh]) OR (Song)) OR (Melody, Vocal)) OR (Vocal Melody)) OR (Vocal Melodies)) OR (Melodies, Vocal)) OR (Songs)) AND (“Infant, Premature”[Mesh])) OR (Infants, Premature)) OR (Premature Infant)) OR (Preterm Infants)) OR (Infant, Preterm)) OR (Infants, Preterm)) OR (Preterm Infant)) OR (Premature Infants)) OR (Neonatal Prematurity)) OR (Prematurity, Neonatal)) AND (premature infant pain profile)) OR (PIPP)) AND (painful procedures)) AND (randomized controlled trial). The reference lists of retrieved studies and relevant reviews were hand-searched, and the process mentioned above was repeatedly performed to ensure the inclusion of all eligible studies. The inclusion criteria were as follows: (1) RCTs, (2) studies in which the patients were preterm infants, (3) studies in which a music intervention was used to relieve pain and discomfort for participants, (4) studies in which a music intervention group was compared with a control group, and (5) full-text studies with sufficient data for extraction for further analysis. Studies written in all languages were included.

### 2.2 Data extraction and outcome measures

The data were independently and separately extracted by two investigators. The following baseline information was extracted from the original studies: first author, publication year, number of patients, gestational age and sex distributions, evidence level, detailed intervention method and duration. Any discrepancies were resolved by consensus. The primary outcomes were the PIPP score during the procedure (absolute change in the PIPP during the procedure, PIPP score during the procedure) and PIPP after the procedure (absolute change in the PIPP score after the procedure, PIPP score after the procedure). The secondary outcomes were the mean SaO_2_, peak heart rate and blood cortisol concentration.

## 3 Results

### 3.1 Literature searches, study characteristics, and quality assessment

**Figure 1:**
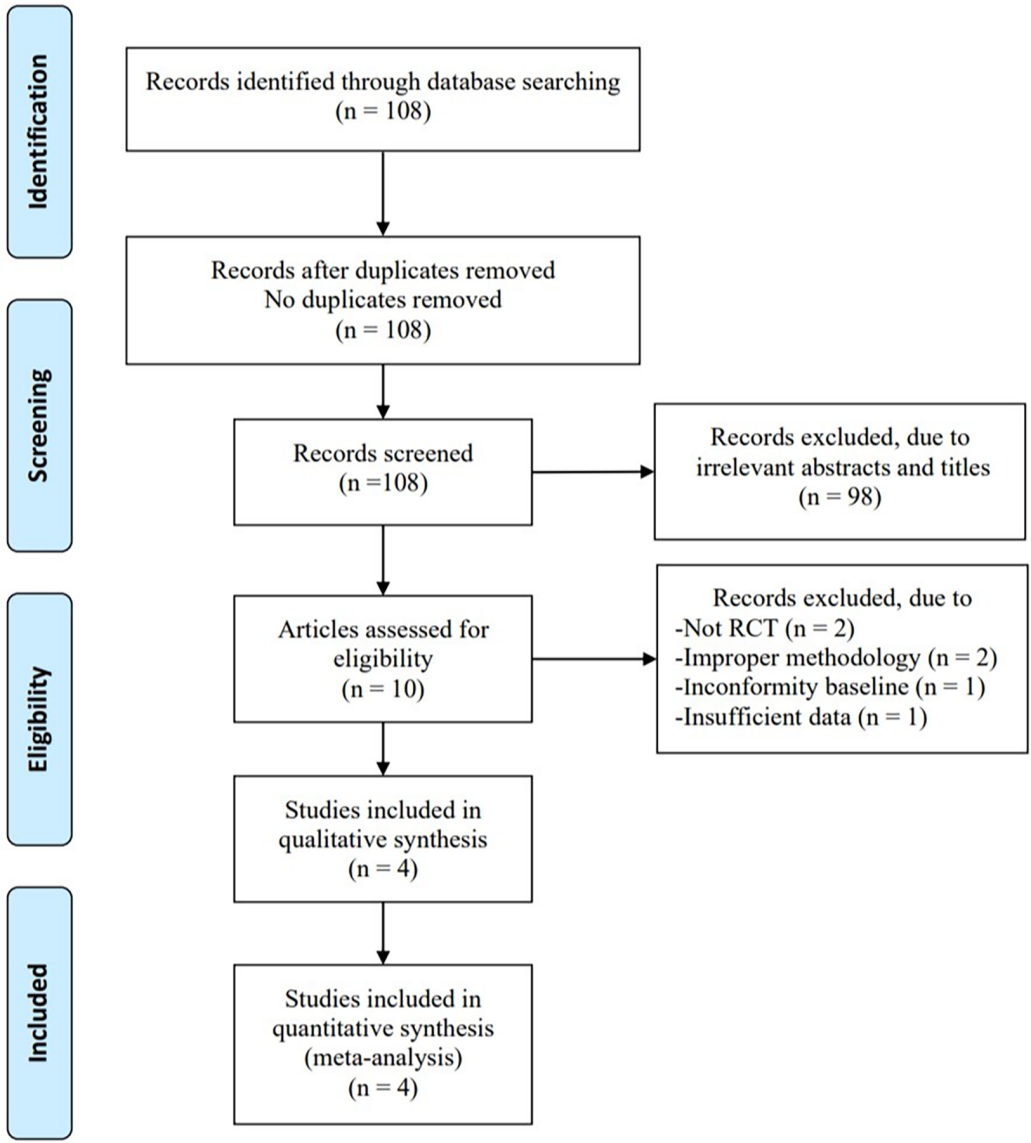
Flow diagram of the study search and selection process.

**Figure 2:**
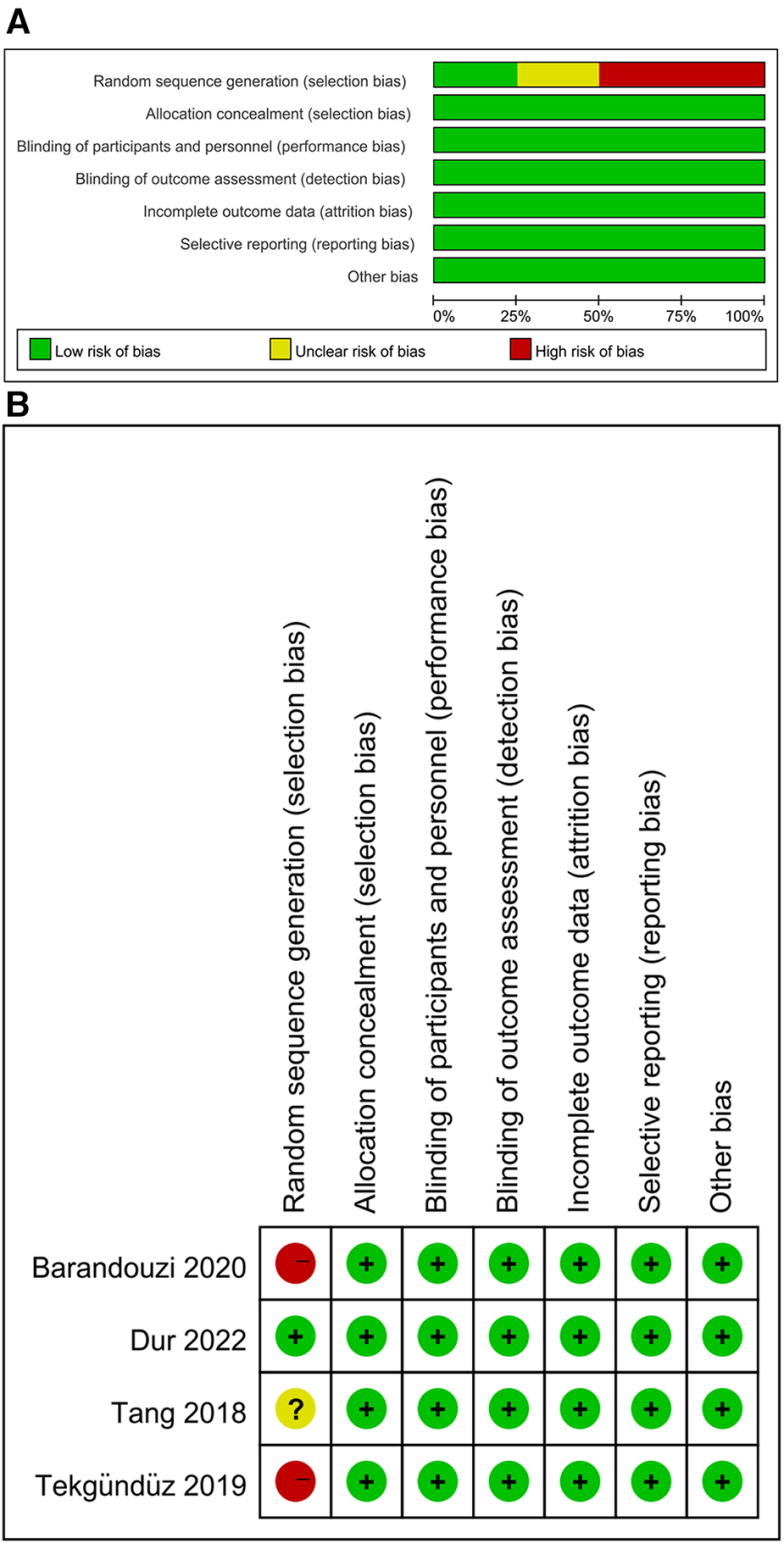
Review author's judgement about each risk of bias item as percentages across all included studies.

In total, 108 articles were initially identified from the databases. After removing duplicates, 108 articles were retained. A total of 98 studies were excluded from our study due to unrelated abstracts and titles. We also excluded two studies that were not RCTs, one study that presented insufficient data, two studies that reported an improper methodology, and one study that presented a nonconformity at baseline. Ultimately, four RCTs satisfied the inclusion criteria and were included in this meta-analysis. The article selection process was performed in accordance with the PRISMA statement (Figure 1). The baseline characteristics of the 4 included studies (23–26) are shown in Table 1. Four studies compared a music intervention to no special intervention. Lullaby music was used in three groups (23, 24, 26), and classical music was used in one group (25). The involved painful clinical procedures included PICC placement (24), nasal continuous positive airway pressure (NCPAP) (23), venipuncture (26) and ROP screening (25). There were no statistically significant differences in patient baseline characteristics. The music duration varied according to the specific procedure. All the studies in our meta-analysis were published between 2018 and 2022, and the total sample size was 252. Two reviewers independently assessed the methodological quality of each study based on the revised Cochrane Risk of Bias tool for RCTs (27). The quality assessment considered seven aspects (random sequence generation, allocation concealment, blinding of participants or personnel, blinding of the outcome assessment, incomplete outcome data, selective reporting, and other bias), with each aspect rated as low, high, or unclear in terms of risk of bias. We used Review Manager (RevMan) software 5.3 to construct a risk of bias graph and summary (Figure 2).

**Table 1:** Characteristics of included studies.

				Music treatment group					Control group				
No	Author	Year	Procedure	Number (n)	Sex (male/female)	(Mean ± SD)	Birth weight (Mean ± SD)	Intervention	Number (n)	Sex (male/female)	Gestational (Mean ± SD)	Birth weight (Mean ± SD)	Intervention
1	Tang	2018	PICC	30	16/14	32.57 ± 1.76	2,185 ± 165	Lullaby and nursery rhymes with50–60 dB was played 10 min before PICC puncture until 10 min after the procedure by an MP4-player with small mobile speaker which placed 30 cm from the infants’ ears.	30	16/14	32.57 ± 1.83	2,212 ± 172	Bed rest
2	Tekgündüz	2019	NCPAP nursing	35	19/16	31.57 ± 3.18	1,774.14 ± 647.74	Lullaby with 50–60 dB was played from the reinsertion of the tracheal tube until the intervention finished by an CD speaker located around 30 cm away from the infants’ heads.	37	19/18	30.26 ± 3.54	1,460.67 ± 684.91	Routine application
3	Barandouzi	2020	venipuncture	30	14/16	34 ± 1.41	1,987 ± 367.86	Lullaby music with 40–50 dB was played 2 min before the venipuncture for 10 min via headphone; received 0.5 ml sterile water via syringe on the anterior portion of infants’ tongue	30	17/13	33.86 ± 1.35	2,012 ± 352.83	Had headphones without music, received 0.5 ml sterile water via syringe on the anterior portion of infants’ tongue
4	Dur	2022	ROP examination	30	13/17	29.43 ± 2.88	1,312.30 ± 404.07	Classic music with 55 dB was played 1 min before PICC puncture until 1 min after the examination via loudspeaker.	30	14/16	29.03 ± 2.63	1,311.33 ± 378.37	swaddled and laid onthe examination table

## 4 Primary outcomes

### 4.1 PIPP score during the procedure

#### 4.1.1 Absolute change in the PIPP score during the procedure

**Figure 3:**
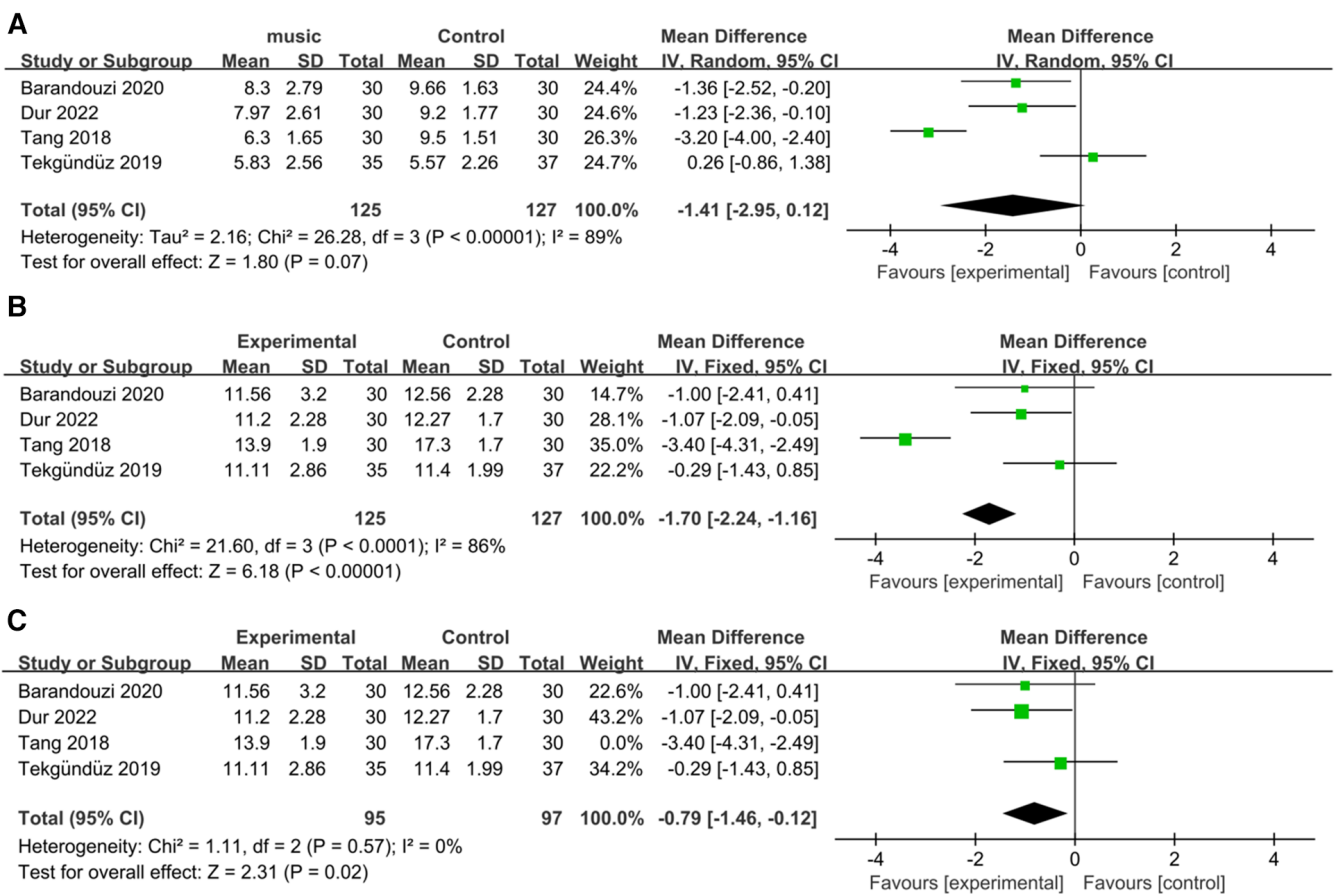
Forest plot for the meta-analysis of PIPP during procedure. (**A**) PIPP absolute change. (**B**) PIPP scores and (**C)** after PIPP scores sensitivity analysis.

All the studies examined the PIPP scores before and during painful procedures. Our results revealed that music did not significantly change the score in the intervention group compared to the control group (RR = −1.41; 95% CI = −2.95−0.12, *p* = 0.07), and there was significant heterogeneity [*I*^2 ^= 89%, *p* < 0.00001; Figure 3A].

#### 4.1.2 PIPP score during the procedure

The PIPP scores significantly differed between the music intervention group and the control group (RR = −1.70; 95% CI = −2.24–−1.16, *p *< 0.00001), and there was significant heterogeneity [*I*^2 ^= 86%, *p *< 0.0001; Figure 3B]. After removing the Tang et al. study, the heterogeneity became nonsignificant [*I*^2 ^= 0%, *p* = 0.57; Figure 3C], and the overall effect of music remained significant (RR = −0.79; 95% CI = −1.46–−0.12, *p* = 0.02).

### 4.2 PIPP score after the procedure

#### 4.2.1 Absolute change in the PIPP score after the procedure

**Figure 4:**
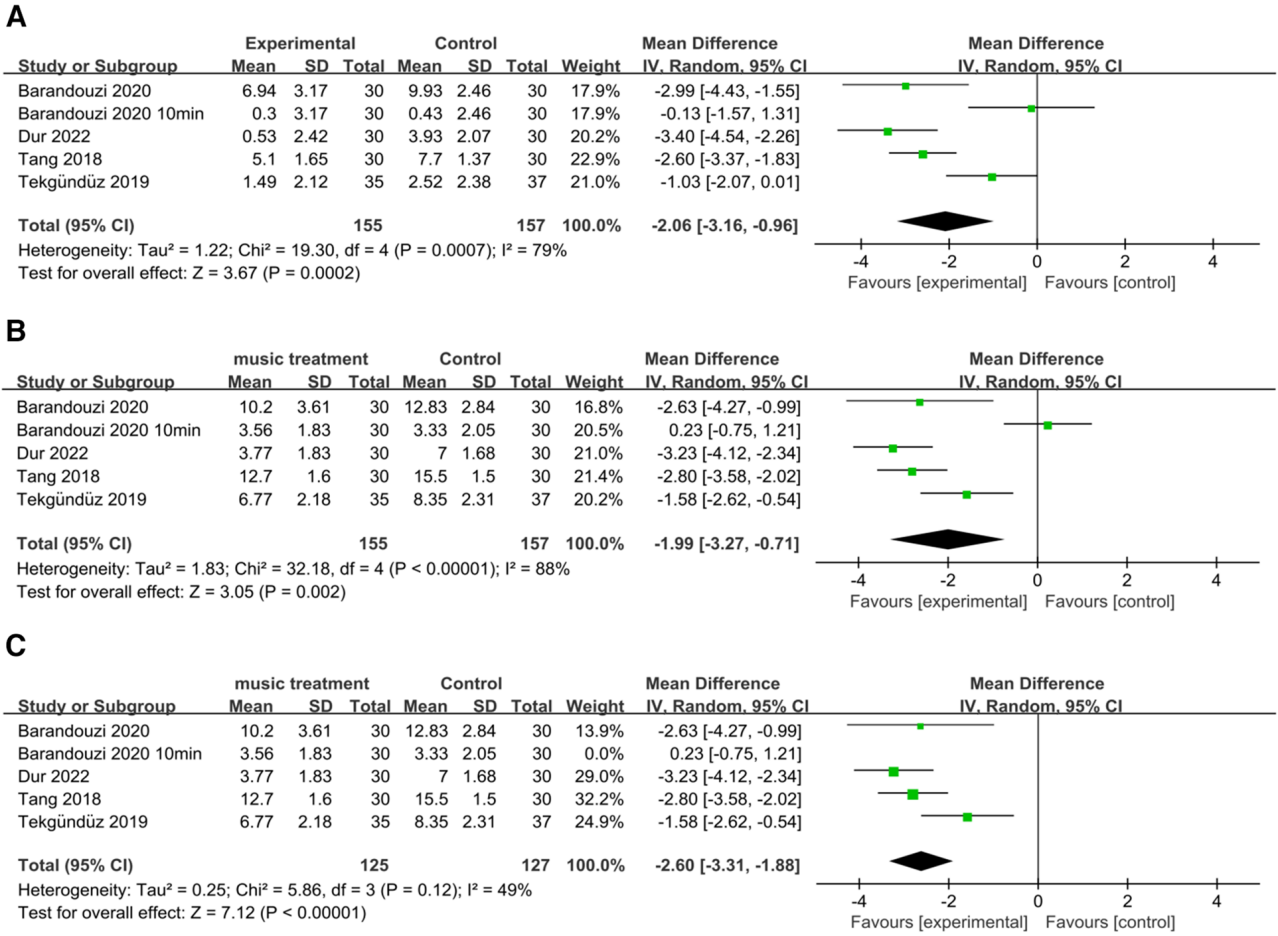
Forest plot for the meta-analysis of PIPP after procedure. (**A**) PIPP absolute change. (**B)** PIPP scores and (**C**) after PIPP scores sensitivity analysis.

Four studies examined the PIPP scores before and after painful procedures. The score change significantly differed between the music intervention group and the control group (RR = −2.06; 95% CI = −3.16–−0.96, *p* = 0.0002), and there was significant heterogeneity [*I*^2^ = 79%, *p* = 0.0007; Figure 4A].

#### 4.2.2 PIPP score after the procedure

Our results revealed a significant difference in the scores of the music intervention group compared with those of the control group (RR = −1.99; 95% CI −3.27–−0.71; *p* = 0.002), and there was significant heterogeneity [*I*^2^ = 88%, *p* < 0.00001; Figure 4B]. After removing the study by Barandouzi et al. in which data was collected 10 min after the procedure, the heterogeneity decreased (*I*^2 ^= 49%, *p* = 0.12), and the overall effect of music remained significant [RR = −2.60; CI = −3.31–−1.88, *p* < 0.00001; Figure 4C].

## 5 Secondary outcomes

### 5.1 Mean SaO_2_ during/after the procedure

**Figure 5:**
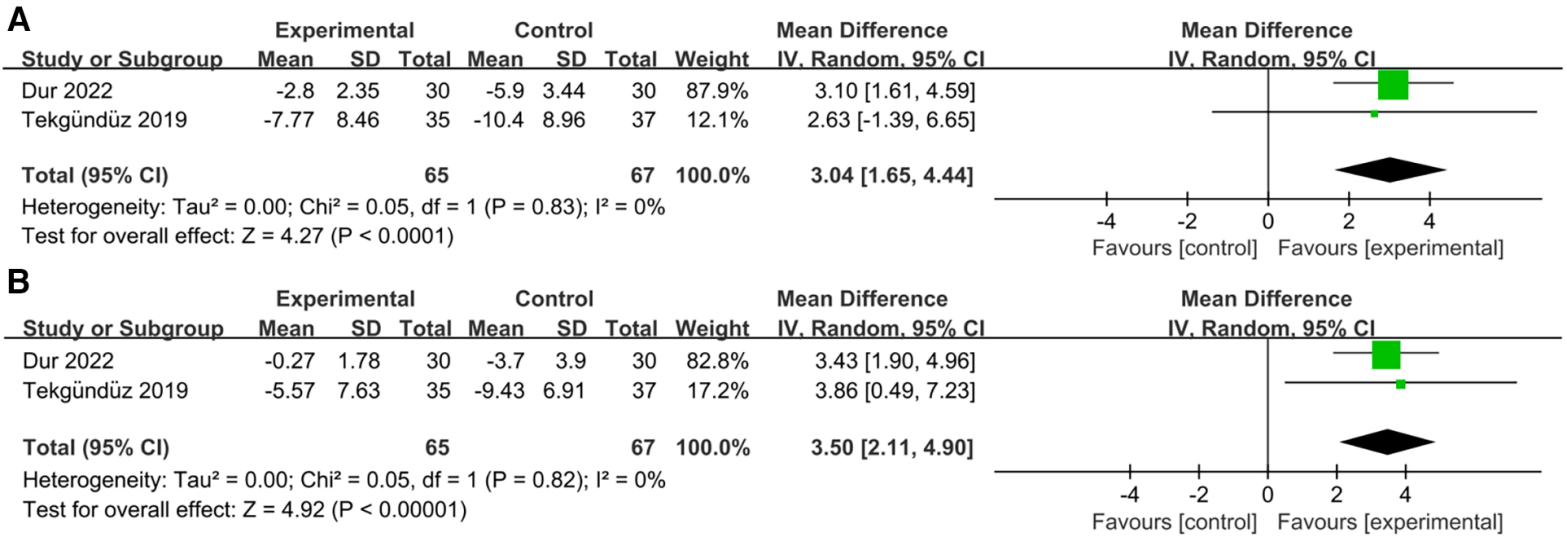
Forest plot for the meta-analysis of mean SaO_2_. (**A**) mean SaO_2_ during procedure and (**B**) mean SaO_2_ after procedure.

Two studies examined data relating to the mean SaO_2_ (23, 25). For the mean SaO_2_, our meta-analysis indicated that there were significant differences in the duration of music during [RR = 3.04, 95% CI = 1.64–4.44, *p* < 0.0001; Figure 5A] and after the procedure [RR = 3.50, 95% CI = 2.11–4.90, *p* < 0.00001; Figure 5B]. There was also nonsignificant heterogeneity (*I*^2 ^= 0%, *p* = 0.83; *I*^2 ^= 0%, *p* = 0.82). Overall, music improved the blood oxygen saturation of preterm infants not only during but also after painful procedures.

### 5.2 Peak heart rate during/after the procedure

**Figure 6:**
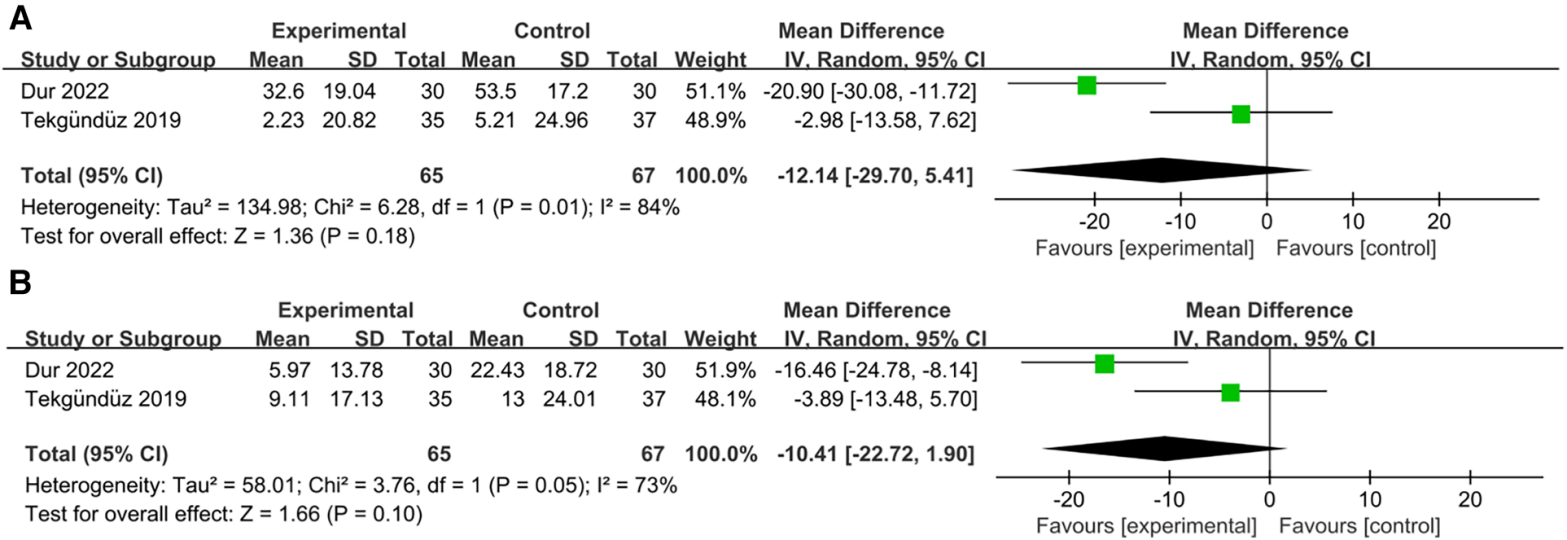
Forest plot for the meta-analysis of peak heart rate. (**A**) peak heart rate during procedure and (**B**) peak heart rate after procedure.

Two studies examined changes in the peak heart rate (23, 25). Our results demonstrated that the music intervention group did not show a significant decrease in the peak heart rate during the procedure compared to the control group [RR = −12.14; 95% CI = −29.70–5.41 *p* = 0.18; Figure 6A], and there was significant heterogeneity (*I*^2 ^= 84%, *p* = 0.01). Additionally, a comparison of the statistical data after the procedure showed a negative correlation [RR = −10.41; 95% CI = −22.72–1.90 *p* = 0.10; Figure 6B], with significant heterogeneity (*I*^2 ^= 73%, *p* = 0.10).

### 5.3 Blood cortisol concentration

One study (24) analyzed the blood cortisol concentration as a stress level index. The results showed that the cortisol concentration was significantly increased in the control group, but no obvious increase was observed in the music intervention group.

## 6 Discussion

Increasing evidence has shown that nonpharmacological analgesic methods are essential components of all effective interventions designed for managing procedural pain in preterm infants. However, there is no comprehensive understanding of the effect of music interventions on pain control and its physiological parameters. Considering that the PIPP has been validated to be a reliable assessment of pain in premature infants (28, 29), we performed this PIPP-centered meta-analysis to evaluate the analgesic effects of music and obtain higher-level evidence.

Our results showed that music had a nonsignificant effect on reducing the absolute change in the PIPP score during invasive procedures. However, due to the high heterogeneity (*I*^2 ^= 89%) and the lack of clarity regarding the specific duration of the intervention may have led to unreliable conclusions. Furthermore, music can significantly reduce pain during painful procedures, and the heterogeneity decreased precipitously from 86% to 0% after removing the study by Tang et al. (24) due to its use of a different methodology. In their study, compared to other studies, music was played for a longer duration (10 min) before the start of the invasive procedure. Moreover, music had a significant effect on pain reduction at the end of the invasive procedure according to the PIPP score after the painful procedure. By comparing the results obtained during and after the operation, the importance of the duration of music before the start of the procedure was reemphasized. Thus, future studies are needed to verify the analgesic effect of playing music for a long period before painful procedures and explore a related standard scheme. According to our results and the findings of previous studies, the analgesic effect of music is understood, and music interventions have great potential for application in clinical practice to ease the pain of preterm infants, reduce the duration of invasive procedures, decrease the use of narcotic and analgesic drugs, and avoid the long-term effects of repetitive, acute episodic pain. Questions for future research remain. The optimal duration and amplitude of music therapy for pain relief still have yet to be determined. There are no studies that aimed to define the best choice of music for music therapy. To our knowledge, the aspect of overstimulation has not been studied. If possible, we strongly recommend that more experts in the field of music therapy focus on and participate in preterm infant acute episodic pain alleviation projects, assisting clinicians and nurses in clinical decision making. Admittedly, we should emphasize that as a supplementary measure, the pain-relieving effect of music is limited because the pain category was unchanged.

Our results also indicated that music could significantly reduce pain levels during daily painful procedures. First, as an auditory stimulus, music not only provides a distraction but also has a potential effect on modulating perception by reducing delta-band activity in the cingulate gyrus and increasing gamma-band activity in somatosensory brain structures at different pain processing stages (30), thereby helping relieve painful responses. Moreover, studies have suggested that the analgesic effects of music can be partly explained by the endorphin release-induced reduction in the sympathetic nervous system response (31). Second, hearing music can physically reduce disturbing noises from the armamentarium and protect neonates against experiencing negative feelings (32, 33). Third, music can block pain pathways by facilitating sensorial saturation (34).

Improvement in physiological parameters is another indication of stress levels that reflect pain management outcomes. Several studies have demonstrated that music leads to an increase in oxygen saturation levels, as it reduces stress and has positive effects on both the heart rate and rhythm as well as on the respiratory rate (20, 35). Our results further validated this result, as music can increase the blood oxygen saturation of neonates during and after painful procedures. According to a recent systematic review, music/vocal interventions could reduce the heart rate in preterm infants during procedures, and the effect was greater after the procedures (36). Nevertheless, music did not have an obvious advantage in decreasing the peak heart rate in the present study. Blood oxygen saturation is affected by multiple factors, such as the whole respiratory and circulatory system. Therefore, the improvement in blood oxygen saturation may be more meaningful than the decrease in the peak heart rate.

As a recognized stress hormone, cortisol has always been a widely used indicator of stress levels (37). Studies have shown that music exposure can decrease stress hormone levels in preterm neonates in the NICU environment (38). Previous studies have confirmed that music decreases the levels of stress hormones before, during, and after surgery (39–41). However, only one of the four studies measured cortisol levels, and the cortisol concentration in the music intervention group was significantly lower than that in the control group, which further confirmed the stress-relieving effect of music. Therefore, additional studies are needed to further analyze cortisol levels to obtain a more visual representation of the influence of music on the degree of stress alleviation.

In this systematic review and meta-analysis, we first investigated the impact of music on pain management in preterm infants during acute episodic pain. This study has several limitations. First, the sample size in each study was relatively small. Second, the included studies involved diverse invasive operations and different evaluation time points, which contributed to a major part of the heterogeneity. Third, the duration and genre of the music and the approach of the music intervention largely varied in this study, and the related standard is worth investigating in future studies. Additionally, the selected studies involved various durations. Finally, missing and unpublished data also led to bias in the true impact of the music intervention. Thus, robust RCTs with large sample sizes and a standard protocol should be conducted in the future to obtain more accurate data and to verify our results.

## 7 Conclusion

**Figure 7:**
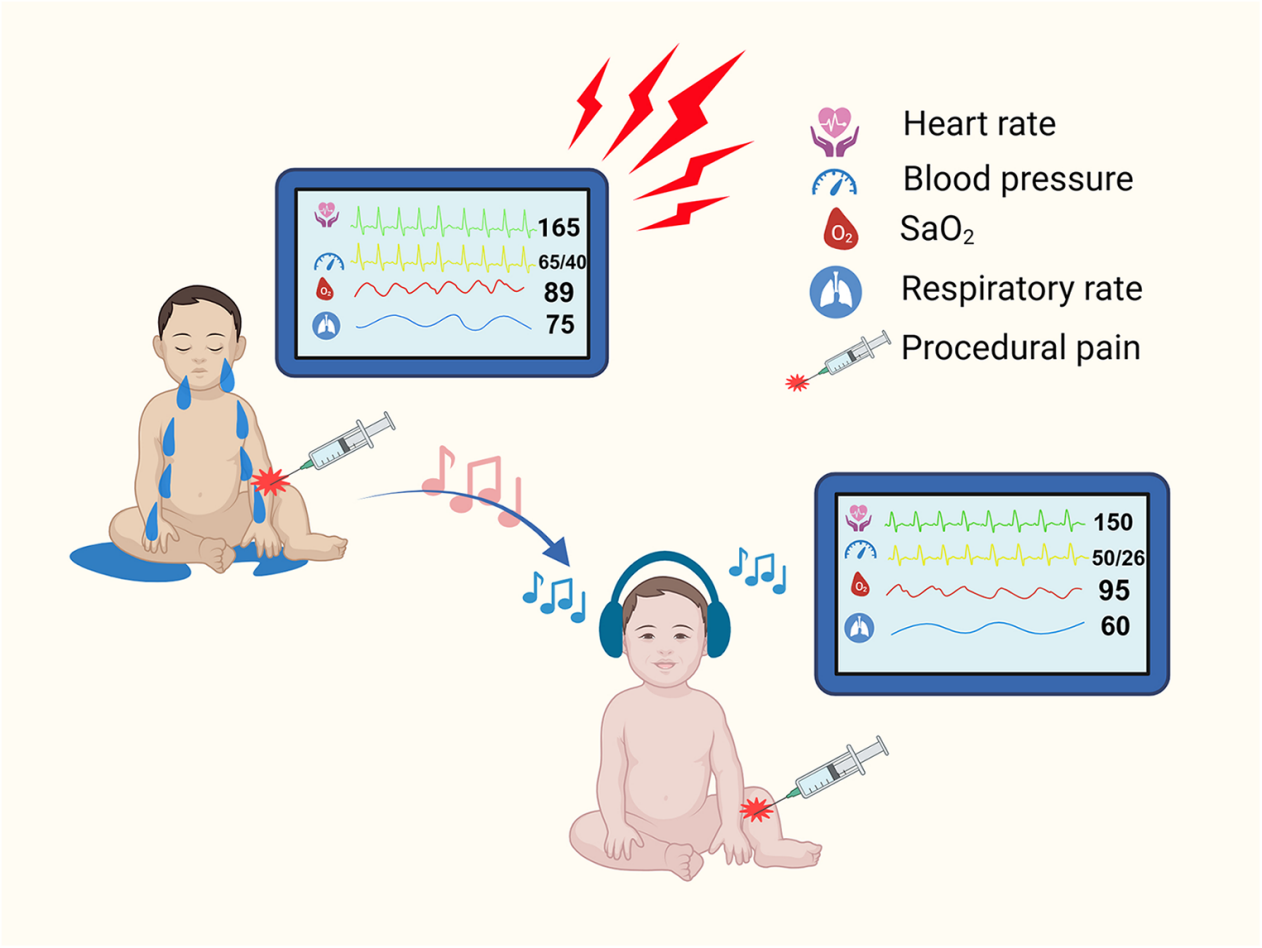
Illustration for the ideal outcome of music intervention of procedural pain management in preterm infants. (Created with BioRender.com.).

In conclusion, this systematic review demonstrated that music is an effective intervention for relieving procedural pain in preterm infants. Our results indicated that music can reduce stress levels and improve blood oxygen saturation. Figure 7 shows the ideal outcome of music for procedural pain management in preterm infants. As a noninvasive, nonpharmaceutical, relatively low-cost intervention that can be applied at the bedside, music has an extremely impressive application in the NICU daily nursing routine. Due to the limitations of the present study, large-scale, prospective RCTs should be performed to validate these results.

## Data Availability

The original contributions presented in the study are included in the article/Supplementary Material, further inquiries can be directed to the corresponding author.
